# Narrowing Down a Major QTL Region Conferring Pod Fiber Contents in Yardlong Bean (*Vigna unguiculata*), a Vegetable Cowpea

**DOI:** 10.3390/genes11040363

**Published:** 2020-03-27

**Authors:** Phurisorn Watcharatpong, Akito Kaga, Xin Chen, Prakit Somta

**Affiliations:** 1 Department of Agronomy, Faculty of Agriculture at Kamphaeng Saen, Kasetsart University, Kamphaeng Saen Campus, Nakhon Pathom 73140, Thailand; watcharatpong_phuriphuri@yahoo.com; 2 Soybean and Field Crop Applied Genomics Research Unit, Institute of Crop Science, National Agriculture and Food Research Organization, 2-1-2, Kannondai, Tsukuba, Ibaraki 305-8602, Japan; kaga@affrc.go.jp; 3 Institute of Industrial Crops, Jiangsu Academy of Agricultural Sciences, 50 Zhongling Street, Nanjing, Jiangsu 210014, China; cx@jaas.ac.cn; 4 Center of Excellence on Agricultural Biotechnology: (AG-BIO/PERDO-CHE), Bangkok 10900, Thailand

**Keywords:** pod fibers, cellulose, hemicellulos, lignin, pod shattering, *beta* glucosidase, MYB26

## Abstract

Yardlong bean (*Vigna unguiculata* (L.) Walp. ssp. *sesquipedalis*), a subgroup of cowpea, is an important vegetable legume crop of Asia where its young pods are consumed in both fresh and cooked forms. Pod fiber contents (cellulose, hemicellulose and lignin) correlates with pod tenderness (softness/hardness) and pod shattering. In a previous study using populations derived from crosses between yardlong bean and wild cowpea (*V. unguiculata* ssp. *unguiculata* var. *spontanea*), three major quantitative trait loci (QTLs), *qCel7.1*, *qHem7.1* and *qLig7.1*, controlling these fibers were identified on linkage group 7 (cowpea chromosome 5) and are co-located with QTLs for pod tenderness and pod shattering. The objective of this study was to identify candidate gene(s) controlling the pod fiber contents. Fine mapping for *qCel7.1*, *qHem7.1* and *qLig7.1* was conducted using F_2_ and F_2:3_ populations of 309 and 334 individuals, respectively, from the same cross combination. New DNA markers were developed from cowpea reference genome sequence and used for fine mapping. A QTL analysis showed that in most cases, each pod fiber content was controlled by one major and one minor QTLs on the LG7. The major QTLs for cellulose, hemicellulose and lignin in pod were always mapped to the same regions or close to each other. In addition, a major QTL for pod shattering was also located in the region. Although there were several annotated genes relating to pod fiber contents in the region, two genes including *Vigun05g266600* (*VuBGLU12*) encoding a beta glucosidase and *Vigun05g273500* (*VuMYB26b*) encoding a transcription factor MYB26 were identified as candidate genes for the pod fiber contents and pod shattering. Function(s) of these genes in relation to pod wall fiber biosynthesis and pod shattering was discussed.

## 1. Introduction

The cowpea (*Vigna unguiculata* L. Walp.) is an economically important crop legume in Africa, America and Asia [1,2,3]. This crop has five cultivar groups/subspecies including *unguiculata* (grain cowpea (also known as black-eye pea)), *biflora*, *texilis*, *melanopthamus* and *sesquipedalis* (yardlong bean (also known as asparagus bean)) [2,4]. Of these five groups, the grain cowpea and yardlong bean are the most popular. The grain cowpea is commonly grown for dry seeds, although sometimes, young pods and young leaves are harvested and consumed as vegetable [5]. The grain cowpea is popularly cultivated in Africa, America and Asia. The yardlong bean is chiefly grown for immature long and tender pods. This crop is popularly cultivated in Asia, especially Southeast Asia and China [5]. Immature pods of yardlong bean are consumed as vegetable in both cooked and fresh forms. It is a source of vitamin, mineral and dietary fibers for human [6,7]. The grain cowpea has been domesticated in Africa [8], whereas the yardlong bean has been domesticated in Asia [9,10,11,12]. 

There are morphological differences between the grain cowpea and the yardlong bean in pod characteristics, including pod length, pod tenderness, pod shattering and pod fibers [5,7,13,14]. The pod length of the yardlong bean is about 30–100 cm, while that of the grain cowpea is about 15–30 cm. The pods of yardlong beans are much softer than those of the grain cowpea. The yardlong bean shows complete loss of pod shattering under any environmental conditions, while the grain cowpea shows some degree of pod shattering in some environmental conditions such as high temperature. The pods of the yardlong bean are less fibrous than those of the grain cowpea. These pod-related traits are genetically correlated. Kongjaimun et al. [14] located quantitative trait loci (QTL) controlling several domestication-related traits using F_2_ and backcross (BC_1_F_1_) populations derived from crosses between yardlong bean accession “JP81610” and wild cowpea (*V. unguiculata* ssp. *unguiculata* var. *spontanea*) accession “TVnu-457”. They found that the largest-effect QTLs for pod length and pod shattering are co-located on linkage group (LG) 7 (corresponding to chromosome 5 of cowpea reference genome) [7]. Later, by using the same F_2_ and BC_1_F_1_ populations, Kongjaimun et al. [7] identified QTLs for pod tenderness and found that the major QTL for this trait is located on the LG7 near to the QTLs for pod length and pod shattering. The difference in pod tenderness and shattering between the yardlong bean and the cowpea is believed to be due to the pod fiber contents [5]. 

By using the F_2_ and BC_1_F_1_ population used by Kongjaimun et al. [7,14], Suanum et al. [5] showed that the contents of pod cellulose, hemicellulose and lignin showed statistically positive and moderate correlation with degree of pod shattering. The authors mapped QTLs for pod fiber content and pod shattering and demonstrated that major QTLs for pod fiber content, *qCel7.1*, *qHem7.1* and *qLig7.1*, are co-localized with major QTL for pod length, pod shattering and pod tenderness on LG7. These QTLs are clustered in the marker interval cp06388 and VR294. In addition, by comparing the linkage maps they used for QTL mapping with a reference whole genome sequence of azuki bean (*Vigna angularis* (Ohwi) Ohwi and Ohashi) [15], they suggested that the gene encoding transcription factor MYB46 is a candidate gene for pod fiber contents and pod shattering. Recently, Lo et al. [16] identified QTLs for the domestication of cowpea using a recombinant inbred line population developed from a cross between grain cowpea breeding line “IT99K-573-1-1” and wild cowpea accession “TVNu-1158”. They detected two significant QTLs, *CPshat3* on chromosome 3 and *CPshat5* on chromosome 5, for pod shattering. These QTLs explained 37.69% and 30.27% of the phenotypic variation, respectively. Interestingly, no QTL for pod length detected on the chromosome 5 where QTL for shattering located onto. This result is contrast to the results reported by Kongjaimun et al. [7] and Suanum et al. [5], who showed localization of QTLs for pod length and pod shattering on the cowpea chromosome 5. Very recently, by employing fine mapping in combination with whole-genome sequencing, Takahashi et al. [17] identified *Vigun05g27350* (*MYB26b*) as the gene responsible for pod shattering (shattering vs. none shattering) in cowpea and azuki bean. 

We are interested in the role(s) of pod fiber content in pod shattering and pod tenderness. In this paper, we report narrowing down the QTL region controlling pod fiber content in the yardlong bean. The objective of this study is to identify candidate gene(s) controlling the pod fiber contents in yardlong bean.

## 2. Materials and Methods 

### 2.1. Mapping Population

Three mapping populations were used in this study; F2A, F2B and F2C. They were all developed from hybridization between JP81610 (female parent) and TVnu-457 (male parent). JP81610 is a yardlong bean (*V. unguiculata* ssp. *sesquipedalis*) from Sri Lanka, whereas TVnu-457 is a wild cowpea (*V. unguiculata* ssp. *unguiculata* var. *spontanea*) from Mali. A diagram of population development is shown in Supplementary Figure S1. Population F2A was an F_2_ population of 177 previously used to locate QTLs for pod shattering [5,7], pod fiber contents [5] and pod tenderness [14]. Population F2B was an F_2_ population of 309 individuals developed in this study. The F2B population and their parents were grown under field conditions during December 2016 to February 2017 in an experimental field of Kasetsart University, Kamphaeng Saen Campus, Nakhon Patom, Thailand. Spacing between plants was 0.70 m. Population F2C was an F_2:3_ generation of 334 plants derived from a self-pollination of 28 F_2_ plants (10–32 F_3_ plants per F_2_ plant) of the F2B population. These 28 F_2_ plants were selected based on heterozygosity of marker at least one genotype between cp08202 to Van07-SSR12 resulting from QTL mapping of the pod fiber contents. The genotypes of these plants are shown in Supplementary Table S1. The F2C population and the parents were planted during October to December in 2017 in the same field as in the F2B population. The total genomic DNA of each plants of the F2B and F2C populations was extracted from young leaves as per Lodhi et al. [18]. The quality and quantity of the DNA was determined by 1% agarose gel electrophoresis and Nanodrop spectrophotometer (Thermo Fisher Scientific™, USA), respectively. At maturity, dry pods of each plant were harvested for a fiber analysis.

### 2.2. Determination of Pod Fiber Contents

Since the pod fiber content data, including cellulose, hemicellulose and lignin in the F2A were available from previous study [5], the traits were determined only in the F2B and F2C populations. In the F2A population, F_2:3_ lines were sown in randomized complete block design with two replicates under field condition. In each replicate, pods of five plants of each line were harvested and used for fiber analysis. In the F2B population, the dry pods harvested from each F**_2_** plants and the parental plants were determined for fiber contents including cellulose, hemicellulose and lignin. Initially, the pods were dried at 80 °C for 48 h. Then, the pods were ground into the powder and sieved through 1 µm sieve. Two samples/replicates of the sieved powder of 0.5 g of each plant were used for a fiber analysis. Before conducting the fiber analysis, the samples were subjected to near infrared (NIR) spectroscopy using BUCHI NIRFlex N-500 Solids (Switzerland). Each sample was measured for NIR spectrum three times. Subsequently, the samples were determined for neutral detergent fiber (NDF), acid detergent fiber (ADF), and acid detergent lignin (ADL) by fiber bag technology using an ANKOM-200 Fiber Analyzer in combination with a Daisy Incubator (ANKOM Technology, Macedon, NY) following the procedures described by Vogel et al. [19]. After that, ADL and ash were determined as per Suanum et al. [5]. Briefly, the ANKOM bags containing the residual of the ADF procedure were placed in a 3-L Daisy incubator and submerged under 72% H_2_SO_4_. The samples were altered in the incubator for 3 h, washed using hot water for 15 min and in acetone for 10 min, dried in a 105 °C oven for 4 h, and then weighed. Subsequently, the sample bag containing the remaining residual fiber was burnt at 550 °C for 4 h. Then, the resultant ash was weighed. Finally, the amount of NDF, ADF, ADL and ash were used to calculate cellulose, hemicellulose and lignin contents following Suanum et al. [5]. 

In the F2C population, dry pods harvested from each F_3_ plants were determined for the fiber contents using NIR spectroscopy. The preparation of the samples and the NIR spectroscopy were the same as described above. NIR equations for cellulose, hemicellulose and lignin were obtained using 206 samples of the F2B populations. The samples were separated into calibration sets (51 samples) and validation sets (152 samples). The formulae were modified using functions “Smooth Savitzky-Golay 9 points”, “Second Derivative”, “Smoothing and SNV (Standard Normal Variate)” for precision adjustment. NIR equation for cellulose was f(x) = 0.8764x + 4.1513; *R*^2^ = 0.7992; BIAS = 0.0119. NIR equation for hemicellulose was f(x) = 0.8250x + 1.8343; *R*^2^ = 0.7785; BIAS = 0.2663. NIR equation for was lignin was f(x) = 0.6781x + 5.8806; *R*^2^ = 0.7440; BIAS = 0.1327. 

### 2.3. Fine Mapping for the Pod Fiber Contents 

Since the major QTLs controlling cellulose, hemicellulose and lignin were mapped between SSR markers cp06388 and VR294 on LG7 [5], 80 SSR markers from cowpea (24 markers) and azuki bean (56 markers) residing around this genome region were developed to finely map the QTLs for the traits (Supplementary Table S2). In brief, primer sequences of the markers cp06388 and VR294 were BLASTed to reference whole genome sequence of cowpea (Vigna unguiculata v.1.1; https://phytozome.jgi.doe.gov/pz/portal.html#!info?alias=Org_Vunguiculata_er) [20] and azuki bean (https://viggs.dna.affrc.go.jp) [15] and searched for SSRs using SSRIT [21]. Primers for the 82 SSRs were then designed using Primer3 [22]. In addition, two insertion/deletion (InDel) markers were developed from our in-house transcript sequence data of cowpea. These markers were screened for polymorphism between the parents and those showing polymorphism were used to analyze DNA of all the three population. The marker analysis (polymerase chain reaction (PCR) and gel electrophoresis and DNA band visualization) were the same as described by Somta et al. [13] and Kongjaimun et al. [23].

### 2.4. Fine Mapping of Pod Shattering

Since a previous study using population F2A by Suanum et al. [5] revealed co-localization between major QTLs for pod fiber contents and pod shattering on LG7, fine mapping of the QTLs for pod fiber contents as well as pod shattering of the F2A population was conducted using additional genetic markers in the present study. Briefly, the pod shattering was evaluated in the F_2:3_ lines grown under field conditions by Suanum et al. [5]. Pod shattering of each F_2:3_ plant was evaluated by visual scoring using a scale of 1 to 5; 1 = no shattering, 2 = pod valves slightly opened, 3 = pod valves opened all along the pod length with minor or without twist, 4 = pod valves opened all along the pod length with moderate twist, and 5 = pod valves opened and twisted all along the pod length. Pod shattering data of the population F2A is shown in Supplementary Figure S2.

### 2.5. Linkage Map and QTL Analyses

A linkage map for each population was constructed from the polymorphic SSRs using QTL IciMapping 4.2 software [24]. Grouping of the markers was done with a minimum logarithm of the odds (LOD) of 3.0. Markers were ordered based on their position on the cowpea reference genome [20]. Recombination frequencies were converted into genetic map distance (centimorgan; cM) using Kosambi’s mapping function [25]. 

Location of the QTL conditioning cellulose, hemicelluloses and lignin content in pod was determined by inclusive composite interval mapping (ICIM) [26] using the same software as for the linkage analysis [24,27]. The significant LOD score threshold for the QTLs of each trait was determined by running a 10,000 permutations test at *P* = 0.001. ICIM was carried out at every 1.0 cM.

## 3. Results

### 3.1. Segreation of Pod Fiber Content in F2B and F2C Population

**Figure 1:**
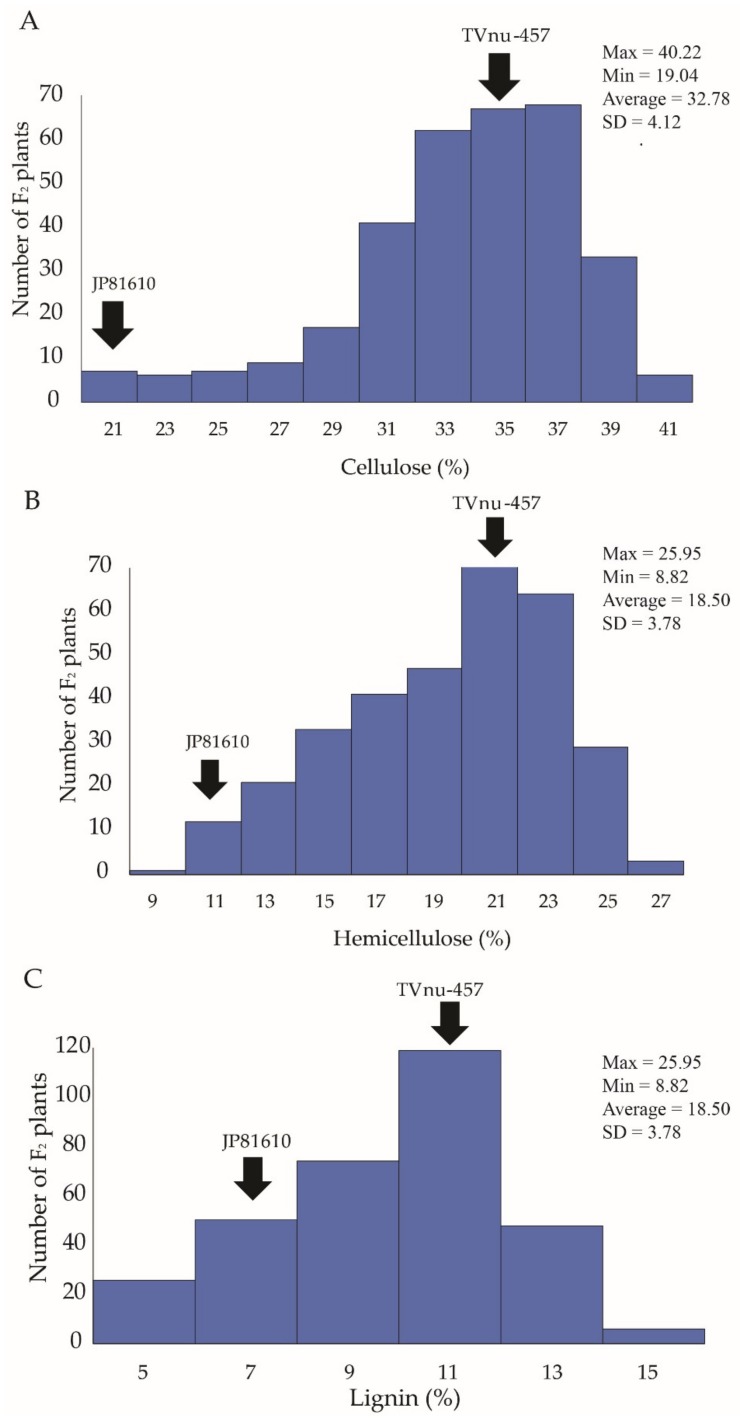
Frequency distribution of cellulose (**A**), hemicellulose (**B**) and lignin (**C**) content of dry pod in F_2_ population F2B of the cross JP81610 × TVnu-457. The fibers were determined by the fiber bag technology method.

**Figure 2:**
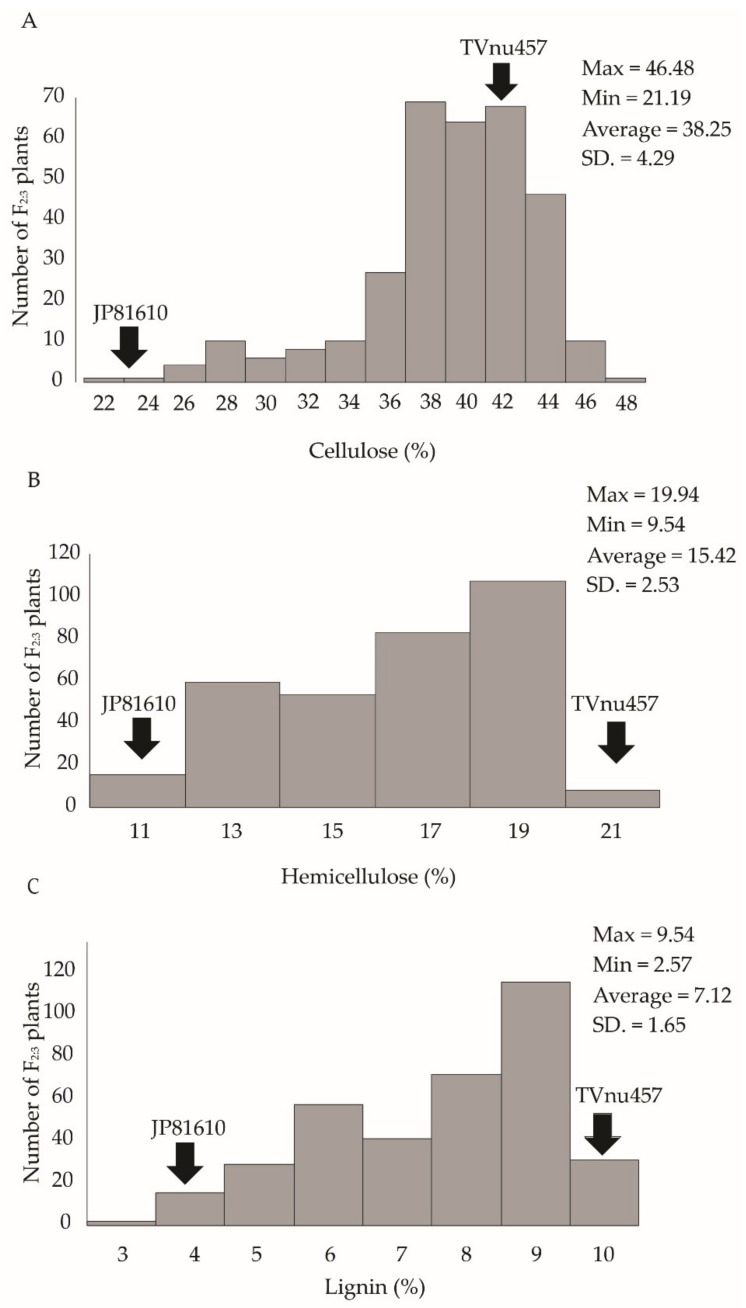
Frequency distribution of cellulose (**A**), hemicellulose (**B**) and lignin (**C**) content of dry pod in F_2:3_ population F2C of the cross JP81610 × TVnu-457. The fibers were determined by the NIR microscopy method.

In the F2B population, pod cellulose contents varied between 19.04% and 40.22% with a mean of 32.78%. Pod hemicellulose contents ranged from 8.82% to 25.96% with a mean of 18.50%. Pod lignin contents were between 3.76% and 14.02% with a mean of 8.78%. The frequency distribution of all the three fibers showed a continuous segregation but skewed towards the wild cowpea (Figure 1). Transgressive segregation to both directions was observed for all the fiber traits (Figure 2). 

In the F2C population, pod fiber contents were measured using NIR microscopy. Pod cellulose contents varied between 21.19% and 46.48% with a mean of 38.25%. Pod hemicellulose contents varied from 9.54% to 19.94% with a mean of 15.42%. Pod lignin contents ranged from 2.57% to 9.54% with a mean of 7.12%. Similarly to the F2B population, all the fibers showed continuous segregation (Figure 2). 

### 3.2. Fine Mapping of QTL for Pod Fiber Content and Pod Shattering

New SSR and InDel markers were developed to fine map major QTLs for cellulose, hemicelulose and lignin. Among the 82 markers developed, only nine of them (three from azuki bean and six markers from cowpea) including Van07-SSR12, Van07-SSR48, Van07-SSR54, cp05951, cp08202, Vu-SSR01-2, VU05-GHIndel, Vu-SSR04, Vu-SSR11 showed clear polymorphism between parents. These markers were used for fine mapping QTLs for pod fiber contents.

**Figure 3:**
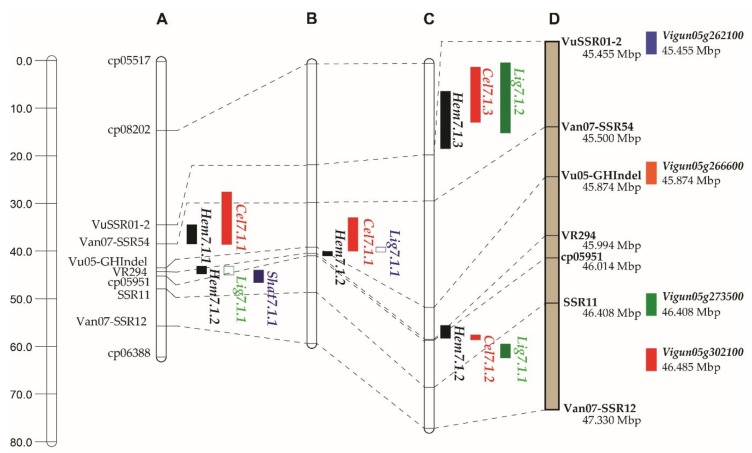
Location of QTLs for cellulose, hemicellulose, and lignin contents in pod and pod shattering identified on linkage group 7 of populations F2A (**A**), F2B (**B**) and F2C (**C**) derived from a cross between JP81610 (yardlong bean) and TVnu-457 (wild cowpea) (see Materials and Methods Section for details). The QTLs were identified by inclusive composite interval mapping. The relationship between the QTLs on linkage maps (A, B and C) and candidate genes on the physical map is also shown (**D**).

In the population F2A, the newly six polymorphic markers together with four markers, previously localized with QTLs for pod fiber contents and pod shattering on LG7 by Suanum et al. [5]) were used to construct a new LG7 for fine mapping. The linkage analysis showed that all the markers were clustered in the same LG7. The new LG7 was 62.0 cM in length (Figure 3A). QTL analysis using this new LG by ICIM detected five QTLs in total; four major QTLs for cellulose, hemicellulose, lignin and pod shattering, and one minor QTL for hemicellulose (Table 1 and Figure 3A). The major QTLs for hemicellulose, lignin and pod shattering were mapped to the same position, which was 44.09 cM between markers cp05951 and VR294. In contrast, the major QTL for cellulose was located far apart from other major QTLs, mapped at 34.30 cM. The major QTLs for cellulose, hemicellulose, lignin and pod shattering accounted for 14.51%, 51.32%, 47.28% and 32.12% of the trait variation, respectively. The minor QTL for hemicellulose was mapped near to the major QTL for cellulose, about 1.2 cM apart. The QTL accounted for about 6% of the total trait variation. This QTL expressed nearly to zero additive effect, but a high dominant effect.

In the popultion F2B, the linkage analysis showed that the eight polymorphic markers grouped together. This linkage group was 58.7 cM in length. ICIM detected three QTLs for pod fiber contents; one each for cellulose, hemicellulose and lignin (Table 2 and Figure 3B). Although the QTLs of these traits were mapped to different marker intervals, they were clustered in a region of about 5.0 cM between markers Van07-SSR54 and cp05951. These QTL accounted for 11.31%, 33.31%, and 58.14% of the trait variation, respectively. 

In the population F2C, the linkage analysis showed that the eight polymorphic markers clustered in the same linkage group with a length of 76.6 cM (Figure 3C). ICIM identified six QTLs for pod fiber contents; two each for cellulose, hemicellulose and lignin (Table 3 and Figure 3C). The major QTLs for these traits were mapped near to one another in a 3-cM region, covering by markers VU05-GHIndel, VR294, cp05951 and Vu-SSR11. The major QTLs for these fibers accounted for 33.14%, 39.10%, and 57.18% of the total trait variation, respectively. The minor QTLs for these traits were mapped to the same marker interval, cp08202 and VUSSR01-2. They explained 10.77%, 5.23%, and 6.57% of the trait variation, respectively.

## 4. Discussion

In legume crops, non-shattering of pods is one of the most important traits that has been selected during domestication by humans. The selection for non-shattering of pods is beneficial for reduced yield loss and ease of harvest. In cowpea/yardlong beans, selection for non-shattering is accompanied by edibility (pod softness/tenderness) of immature pods [5,14,17] and increased seed size [17], although it is unknown whether these traits are controlled by the same gene or tightly linked genes. In this study, we finely mapped major QTLs for pod fiber content in yardlong bean on LG7 using three segregating populations derived from the same parents. In most cases, each type of fiber was controlled by one major and one minor QTL on the LG7. In the same population, the major QTLs for cellulose, hemicellulose and lignin in pod were always mapped to the same regions or close to each other (Table 1, Table 2 and Table 3 and Figure 3A–C). In different populations, the major QTLs for different fibers were always detected in the region of markers Van07-SSR54, Vu05-GHIndel, VR294, cp05951, and Vu-SSR011 (Table 1, Table 2 and Table 3 and Figure 3A–C), while the minor QTLs were in general mapped to different regions (Table 1, Table 2 and Table 3 and Figure 3A–C). 

Based on the current cowpea reference genome sequences [20], physical distance between Van07-SSR54 and Vu-SSR011 is 0.545 Mbp (Figure 3D). There were 91 annotated genes in this region. Among those genes, genes encoding for beta-glucosidases and MYB26 (Figure 3D) are found to be related to cell wall biosynthesis. Surprisingly, fifteen genes encoding for beta glucosidases existed in the region, 14 of which are in tandem. Beta-glucosidase is a glycoside hydrolase enzyme and is involved in the phenylpropanoid pathway that leads to lignin biosynthesis. Moreover, beta-glucosidase is a cellulase enzyme playing a part in metabolism of cell wall polysaccharides in both prokaryotes and eukaryotes. It is involved in plant cell wall development from cell wall degradation pathway [28]. In barley (*Hordeum vulgare* L.), a seed-specific beta-glucosidase is accumulated to high levels during late seed development and participates in endosperm cell wall degradation during germination [29]. Recently, a study in cotton (*Gossypium hirsutum* L.) revealed that a gene, *GhBG1A*, encoding *beta*-glucosidase plays role in cotton fiber elongation and secondary cell wall cellulose deposition [30]. The overexpression of the *GhBG1A* at the fiber elongation stage repressed fiber length but promoted cellulose biosynthesis resulting in thicker fiber cell wall [26]. Based on gene expression analysis in two near-isogenic lines (NILs) showing contrast pod tenderness (soft vs. hard) derived from the cross between JP81610 and TVnu-457 revealed that *Vigun266600* (*VuBGLU12*) encoding for *beta*-glucosidase shows markedly differential expression between the two NILs during stage of pod growth and development (A. Kaga, unpublished data). Interestingly, *VuBGLU12* resides in the 0.545-Mb region controlling pod fiber content and pod shattering (Figure 3). In fact, the indel marker VU05-GHIndel is developed from the transcript sequence of *VuBGLU12*. Thus, the indel polymorphism detected between JP81610 and TVnu-457 by the marker VU05-GHIndel suggests that the VuBGLU12 in these accessions is different. Therefore, *VuBGLU12* is considered as a candidate gene for pod fiber contents in yardlong bean and pod shattering. Additional study is necessary to clarify the association between *VuBGLU12* and fiber contents and shattering of pod in cowpea. 

MYB26 is a transcription factor related to cell wall biosynthesis. In poplar (*Populus trichocarpa*), PtMYB26 activates lignin biosynthesis genes [31]. In Arabidopsis, AtMYB26 functions as master switches, activating secondary wall biosynthesis in anther endothecium affecting anther dehiscence [32,33] and regulates NAC domain transcription factors NST1 and NST2 that act as master regulator in cell wall biosynthesis [34]. Recently, fine mapping in backcross inbred lines ((JP81610 × TVnu-457) × JP81610) together with whole genome sequencing and gene expression analysis revealed *Vigun05g273500* (*VuMYB26b*) as the gene responsible for pod shattering [17]. The authors showed that a SNP disrupting the junction site of the 1^st^ intron and the 2^nd^ exon of the gene and resulting in premature stop codon in JP81610. *VuMYB26b* was expressed only in pods. The function of *VuMYB26b* is believed to be lignification of pod sclerenchyma tissue (increase pod lignin content and thus pod shattering) [17]. It is worth noting that in these two studies, pod shattering was evaluated by binarizing pod characteristics, visual scoring of pod shattering (shattering vs. non-shattering) and feeling tenderness of pods (hard vs. soft), respectively [16,17]. However, in our study, the SSR marker Vu-SSR11 developed from *VuMYB26b* was always not the best marker showing association with major QTLs for fiber contents and pod shattering (Table 1, Table 2 and Table 3 and Figure 2 and Figure 3). It is worth mentioning that Suanum et al. [5] showed that pod shattering determined by scoring degrees of pod shattering in cowpea/yardlong bean shows the highest correlation with pod hemicellulose content, not pod lignin content [5]. A similar result was reported in the common bean (*Phaseolus vulgaris* (L.)), a legume closely related to cowpea, where *PvMYB26* is one of the genes identified in the major QTL region on chromosome 5 controlling pod shattering but the gene was not the best candidate gene showing association with the trait [35,36]. Parker et al. [35] noted that the most significant marker at this QTL located very near to *PvMYB46*, being only about 22 Kb from the gene. Based on reference genome of azuki bean [15], Suanum et al. [5] suggested that *VuMYB46* (*Vigun05g262100*) is a candidate gene at the major QTLs on LG7 for pod fiber contents and pod shattering in cowpea. MYB46 function as master switches activating secondary cell wall biosynthesis [37]. Although, in the present study, *VuMYB46* was not in the core QTL region controlling cellulose and pod shattering, in the F2A population, a major QTL for cellulose was mapped near to *VuMYB46* (Figure 3D). In *Arabidopsis*, AtMYB46 directly regulates expression of secondary wall-associated cellulose synthase genes (*CESA4*, *CESA7* and *CESA8*) [38]. This suggests *VuMYB46* as another candidate gene involved in the pod fiber contents. In fact, MYB26 is an upstream regulator of MYB46 [34]. Additional study is necessary to determine whether *VuBGLU12*, *VuMYB26* and *VuMYB46* are involved in the pod fiber contents in yardlong bean. Large-effect QTLs controlling pod fiber contents on chromosome 5 of yardlong bean may be synergetic effect of more than one genes that are tightly linked and function in network fashion.

## Figures and Tables

**Table 1:** Locations and effects of QTLs identified for pod fiber contents and pod shattering in F_2:3_ population F2A of the cross JP81610 × TVnu-457 by inclusive composite interval mapping.

QTL Name	Trait	Position	Flanking Marker	LOD	PVE (%)	Add	Dom
*Hem7.1.1*	Hemicellulose	35.30	Vu-SSR01-2–Van07-SSR54	3.75	5.79	0.03	1.68
*Hem7.1.2*	Hemicellulose	44.09	Vu05-GHIndel–VR294	25.30	51.32	−3.50	0.33
*Cel7.1.1*	Cellulose	34.30	Vu-SSR01-2–Van07-SSR54	5.44	14.51	−2.37	0.86
*Lig7.1.1*	Lignin	44.09	Vu05-GHIndel–VR294	22.85	47.28	−1.87	0.39
*Shat7.1.1*	Shattering	44.09	Vu05-GHIndel–VR294	13.20	32.12	−0.68	0.23

**Table 2:** Locations and effects of QTLs identified for pod fiber contents in F_2_ population F2B of the cross JP81610 × TVnu-457 by inclusive composite interval mapping**.**

QTL Name	Trait	Position	Flanking Marker	LOD	PVE (%)	Add	Dom
*Hem7.1.2*	Hemicellulose	40.10	VR294–cp05951	51.48	33.31	−3.23	2.17
*Cel7.1.1*	Cellulose	35.70	Van07-SSR54–Vu05-GHIndel	9.64	11.31	−2.03	1.44
*Lig7.1.1*	Lignin	38.60	Vu05-GHIndel–VR294	57.09	58.14	−2.17	1.53

**Table 3:** Locations and effects of QTLs identified for pod fiber contents in F_2:3_ population of the cross JP81610 × TVnu-457 by inclusive composite interval mapping.

QTL Name	Trait	Position	Flanking Marker	LOD	PVE (%)	Add	Dom
*Hem7.1.3*	Hemicellulose	11.40	cp08202–Vu-SSR01-2	4.46	5.23	−0.65	1.08
*Hem7.1.2*	Hemicellulose	57.19	Vu05-GHIndel–VR294	44.44	39.10	−2.17	0.14
*Cel7.1.3*	Cellulose	7.80	cp08202–Vu-SSR01-2	11.25	10.77	−1.72	1.09
*Cel7.1.2*	Cellulose	58.09	VR294–cp05951	41.00	33.14	−2.93	0.38
*Lig7.1.2*	Lignin	9.30	cp08202–Vu-SSR01-2	6.51	5.47	−0.79	0.72
*Lig7.1.1*	Lignin	60.19	cp05951–Vu-SSR11	72.95	57.68	−2.27	2.31

## Supplementary Materials

The following are available online at https://www.mdpi.com/2073-4425/11/4/363/s1. Figure S1: A diagram depicting development of mapping populations (F2A, F2B and F2C) used in this study. Figure S2: Frequency distribution of pod fiber content (hemicellulose (A), cellulose (B) and lignin (C)) and pod shattering (D) in F2A population (F_2:3_ generation) of the JP81610 × TVnu-457. Figures are drawn using data from the study of Suanum et al. [5] with permission from Springer Nature. Table S1: Marker genotypes of 28 F_2_ plants of F2B population (JP81610 x TVnu-457) selected for generating F2C population (F_2:3_ generation). A, B, H and—represent JP81610 genotype, TVnu-457 genotype, heterozygous and missing data, respectively. Table S2: Characteristics of DNA markers used in this study.
